# Novel corticotropin-releasing hormone receptor genes (*CRHR1* and *CRHR2*) linkage to and association with polycystic ovary syndrome

**DOI:** 10.1186/s13048-023-01159-5

**Published:** 2023-08-05

**Authors:** Mutaz Amin, Nicholas Horst, Rongling Wu, Claudia Gragnoli

**Affiliations:** 1 INSERM, US14-Orphanet, 75014 Paris, France; 2 Department of Biochemistry and Molecular Biology, Faculty of Medicine, Al-Neelain University, Khartoum, Sudan 11121; 3 Division of Endocrinology, Department of Medicine, Creighton University School of Medicine, Omaha, NE 68124 USA; 4 Department of Public Health Sciences, Penn State College of Medicine, Hershey, PA 17033 USA; 5 Department of Statistics, Penn State College of Medicine, Hershey, PA 17033 USA; 6 Molecular Biology Laboratory, Bios Biotech Multi-Diagnostic Health Center, 00197 Rome, Italy

**Keywords:** Corticotropin-releasing hormone receptor, CRHR, Polycystic ovarian syndrome, PCOS, Cortisol, Hypothalamic–pituitary–adrenal axis, HPA-axis, Gene, Infertility, Association, Ovary

## Abstract

**Background:**

Women with polycystic ovarian syndrome (PCOS) have increased hypothalamic–pituitary–adrenal (HPA) axis activation, pro-inflammatory mediators, and psychological distress in response to stressors. In women with PCOS, the corticotropin-releasing hormone (CRH) induces an exaggerated HPA response, possibly mediated by one of the CRH receptors (CRHR1 or CRHR2). Both CRHR1 and CRHR2 are implicated in insulin secretion, and variants in *CRHR1* and *CRHR2* genes may predispose to the mental-metabolic risk for PCOS.

**Methods:**

We phenotyped 212 Italian families with type 2 diabetes (T2D) for PCOS following the Rotterdam diagnostic criteria. We analyzed within *CRHR1* and *CRHR2* genes, respectively, 36 and 18 microarray-variants for parametric linkage to and/or linkage disequilibrium (LD) with PCOS under the recessive with complete penetrance (R1) and dominant with complete penetrance (D1) models. Subsequentially, we ran a secondary analysis under the models dominant with incomplete penetrance (D2) and recessive with incomplete penetrance (R2).

**Results:**

We detected 22 variants in *CRHR1* and 1 variant in *CRHR2* significantly (*p* < 0.05) linked to or in LD with PCOS across different inheritance models.

**Conclusions:**

This is the first study to report *CRHR1* and *CRHR2* as novel risk genes in PCOS. In silico analysis predicted that the detected *CRHR1* and *CRHR2* risk variants promote negative chromatin activation of their related genes in the ovaries, potentially affecting the female cycle and ovulation. However, *CRHR1*- and *CRHR2*-risk variants might also lead to hypercortisolism and confer mental-metabolic pleiotropic effects. Functional studies are needed to confirm the pathogenicity of genes and related variants.

## Background

Polycystic ovarian syndrome (PCOS) has a global prevalence of 5–10% and is the most common endocrine disorder among reproductive age women; it is heterogenous, multifactorial, and complex due to environmental and genetic factors not yet fully elucidated, and increases the risk for obesity and type 2 diabetes (T2D) [1–3].

PCOS decreases self-esteem and quality of life, particularly among infertile women, is associated with chronic stress, which can lead to insulin resistance and inflammation [4], and contributes to high levels of depression and anxiety [5–7], which are linked to impaired stress responses [8, 9]. The hypothalamic–pituitary–adrenal (HPA) axis regulates stress response [10], and women with PCOS have increased HPA-axis activation, pro-inflammatory mediators, and psychological distress in response to stressors [11]. Serum cortisol levels are significantly higher in patients with PCOS [12] and more than half have impaired glucocorticoid sensitivity [13]. Familial clustering, twin studies, susceptibility loci, and risk genes with varying susceptibility and penetrance demonstrate a genetic basis of the disorder [14–16]. As the HPA-axis dysfunction may be due to genetic predisposition [4], genes within the HPA-axis pathway, if impaired, might contribute to PCOS.

Of interest, the corticotropin-releasing hormone (CRH) is secreted by hypothalamic paraventricular neurons in response to stress and drives HPA activation that stimulates adrenal glucocorticoid and androgen secretion [17]. CRH induces an exaggerated HPA response in women with PCOS [18]. CRH mediates its effects through corticotropin-releasing hormone receptor (CRHR) 1 and 2 [19]. Both *CRHR1* and *CRHR2* are expressed in discrete patterns in the brain and peripheral tissues, including skeletal muscle, adrenal glands, pancreas, and adipose, immune, and reproductive tissues [20], and integrate central and peripheral stress responses, including energy balance and metabolism [17, 20]. *CRHR1* and *CRHR2* are expressed on the surface of mammalian ovaries mediating CRH roles in ovulation and steroid biosynthesis [21]. In addition, *CRHR1* and *CRHR2* are expressed on pancreatic islet cells: CRHR1 promotes β-cell proliferation and insulin secretion in a glucose-dependent manner; CRHR2 regulates glucose-stimulated insulin secretion mediated by the ligand urocortin 3; and, both variably respond to HPA-axis activation [22–26]. As PCOS is associated with maladaptive stress-driven HPA activation [11] and HPA dysfunction plays a role in the metabolic and inflammatory pathogenesis of PCOS, including insulin resistance [27], and CRHR1 and CRHR2 are implicated in insulin secretion [22–26], *CRHR1* and *CRHR2* variants may predispose to the mental-metabolic risk for PCOS. Therefore, we aimed to investigate whether *CRHR1* and *CRHR2* variants are in linkage to and/or linkage disequilibrium (i.e., association) with PCOS in Italian families.

## Results

**Fig. 1:**
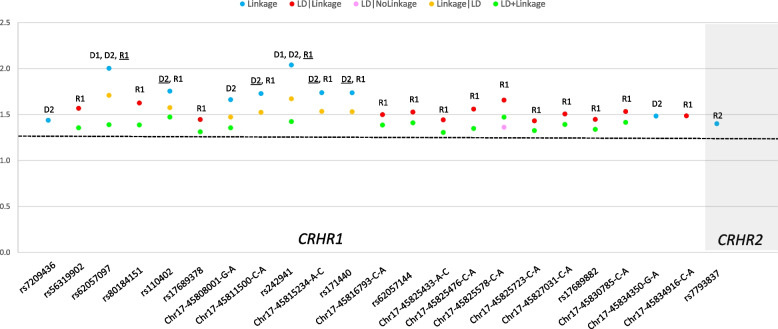
Parametric Analysis Results of Polycystic Ovarian Syndrome (PCOS) *CRHR1* and *CRHR2*-Risk Single Nucleotide Polymorphisms (SNPs). Legend. For each *CRHR1* and *CRHR2*-risk SNPs in PCOS, we present the − log10(P) as a function of the significant (*p* < 0.05) test statistics [(Linkage, linkage disequilibrium (LD)|Linkage, LD|NoLinkage, Linkage|LD, and LD + Linkage)] and per inheritance model. D1: dominant, complete penetrance, D2: dominant, incomplete penetrance, R1: recessive, complete penetrance, R2: recessive, incomplete penetrance. The most significant of each test statistics is underlined

We detected 22 variants in *CRHR1* and 1 variant in *CRHR2* significantly linked to or in LD with PCOS across different inheritance models (*p* < 0.05). Table 1 shows information on the variant, model(s) under which it confers risk, chromosome location, Ref and Alt alleles, putative risk alleles, and whether it falls within an LD block. Figure 1 shows the results of the linkage and LD analyses. All variants were novel and were not associated with any of the PCOS-related traits (i.e., irregular menses, T2D, obesity, insulin resistance, hirsutism, acne, hyperandrogenism, hyperandrogenemia, anovulation, oligoamenorrhea, male-pattern balding, infertility). Three sets of LD blocks in *CRHR1* were identified (Set01, Set02, and Set06) (Table 1).

**Table 1:** Polycystic Ovarian Syndrome (PCOS) *CRHR1* and *CRHR2*-Risk Single Nucleotide Polymorphisms (SNPs)

Gene	Model^a^	SNP	Position	Ref	Alt	Risk Allele	Consequence	LD block	Reported in PCOS?
*CRHR1*	D2	rs7209436	45,792,776	C	T	T	Intronic	Set06	Novel
	R1	rs56319902	45,794,616	C	T	T	Intronic	Set02	Novel
	D1, D2, R1	rs62057097	45,795,918	C	T	C	Intronic	Independent	Novel
	R1	rs80184151	45,801,942	A	G	G	Intronic	Set02	Novel
	D2, R1	rs110402	45,802,681	G	A	A	Intronic	Set06	Novel
	R1	rs17689378	45,804,424	C	T	T	Intronic	Set02	Novel
	D2	Chr17-45,808,001-G-A	45,808,001	G	A	A	Intronic	NA	Novel
	D2, R1	Chr17-45,811,500-C-A	45,811,500	C	A	A	Intronic	NA	Novel
	D1, D2, R1	rs242941	45,815,154	A	C	C	Intronic	Independent	Novel
	D2, R1	Chr17-45,815,234-A-C	45,815,234	A	C	C	Intronic	NA	Novel
	D2, R1	rs171440	45,816,121	G	A	A	Intronic	Independent	Novel
	R1	Chr17-45,816,793-C-A	45,816,793	C	A	A	Intronic	NA	Novel
	R1	rs62057144	45,824,192	A	G	G	Intronic	Set02	Novel
	R1	Chr17-45,825,433-A-C	45,825,433	A	C	C	Intronic	NA	Novel
	R1	Chr17-45,825,476-C-A	45,825,476	C	A	A	Intronic	NA	Novel
	R1	Chr17-45,825,578-C-A	45,825,578	C	A	A	Intronic	NA	Novel
	R1	Chr17-45,825,723-C-A	45,825,723	C	A	A	Intronic	NA	Novel
	R1	Chr17-45,827,031-C-A	45,827,031	C	A	A	Intronic	NA	Novel
	R1	rs17689882	45,829,462	G	A	A	Intronic	Set02	Novel
	R1	Chr17-45,830,785-C-A	45,830,785	C	A	A	Intronic	NA	Novel
	D2	Chr17-45,834,350-G-A	45,834,350	G	A	A	Intronic	NA	Novel
	R1	Chr17-45,834,916-C-A	45,834,916	C	A	A	3’-UTR	NA	Novel
*CRHR2*	R2	rs7793837	30,687,161	A	T	T	Intronic	Independent	Novel

^a^Models: D1: dominant, complete penetrance, D2: dominant, incomplete penetrance, R1: recessive, complete penetrance, R2: recessive, incomplete penetrance; 3’-UTR is the 3’ untranslated region

### In silico findings

All *CRHR1* and *CRHR2* risk variants in our study intersected with a repressed chromatin state in the ovarian tissue and thus confer a potential negative gene expression in ovaries (RegulomeDB) [28].

## Discussion

The corticotropin-releasing hormone receptors are essential components of the HPA axis which mediates the stress response and could potentially be implicated in stress and/or cortisol related pathologies [17]. We recently reported *CRHR2* [29] as novel risk gene in the comorbidity of T2D and major depressive disorder (MDD). In this study, we report the novel linkage and association of the two corticotropin-releasing hormone receptors genes (*CRHR1* and *CRHR2*) with the risk of PCOS in multigenerational Italian families. We identified 22 variants in *CRHR1* significantly linked and in LD with PCOS and 1 variant in *CRHR2* significantly linked to PCOS. Seven of the *CRHR1*-risk variants were in 2 LD blocks previously found to be linked to T2D and MDD in a prior analysis (unpublished results) and the same risk alleles of the variants (rs7209436, rs62057097, rs110402, and rs242941) in our current study were significantly linked and associated with the risk of T2D in the same dataset. PCOS and T2D share several risk traits such as obesity and insulin resistance [30, 31]. Each of these two prevalent conditions could therefore predispose and precede the risk of the other.

Interestingly, the *CRHR1*-risk variants reported in our study were significantly *linked* to PCOS predominantly under the dominant model with incomplete penetrance D2 and were significantly *associated* (or in LD) with PCOS predominantly under the recessive model with complete penetrance R1. This might be explained by a dose-dependent allelic risk since PCOS is a multifactorial complexly inherited condition and the homozygosity of risk alleles under the recessive model (which may occur in homogeneous populations such as the Italian families under study) could potentiate the risk (i.e., association) to PCOS.

The mechanism by which the risk variants could be implicated in the pathogenesis of PCOS remains to be fully elucidated. No transcription factor binding was predicted to be altered by one of the risk alleles upon subsequent in silico analysis. However, all *CRHR1* and *CRHR2* risk variants in our study intersected with a repressed chromatin state in the ovarian tissue, thereby potentially conferring a negative gene expression in ovaries. As both *CRHR1* and *CRHR2* are expressed on the surface of mammalian ovaries and mediate CRH actions on ovulation and steroid biosynthesis [21], this repressed gene activation in the ovaries might impair the signaling essential for the female cycle regulation, steroid synthesis, follicles maturation, and ovulation phase, and contribute to the anovulatory cycles typical of PCOS. However, given that 7 of the *CRHR1*-risk variants are in 2 LD blocks linked to T2D and MDD (unpublished data) and the same PCOS-risk alleles of 4 variants are significantly linked and associated with T2D, we can not a priori exclude that the mental-metabolic contribution risk, at least for these variants, might underlie the PCOS-related maladaptive stress response [11] and the increased blood cortisol levels found in 50% PCOS patients [13, 32], which, as we previously hypothesized [33] and recently reported for *CRHR2* [29], might per se contribute to T2D and MDD as well. Furthermore, T2D and MDD are comorbid with PCOS [34, 35].

However, our present data highlight a direct possible role of *CRHR1* and *CRHR2* risk variants intersecting with repressed ovarian chromatin, thus implying a potential ovarian-specific role of the risk variants. In conclusion, *CRHR1*- and *CRHR2*-risk variants might confer pleiotropic effects, some specific to PCOS, and some related to hypercortisolism, T2D, and MDD.

## Conclusion

This is the first study to report *CRHR1* and *CRHR2* as novel risk genes in PCOS at least in Italian families. Our results should be validated in other ethnicities and functional studies are needed to confirm the pathogenicity of the reported genes and related variants.

## Methods

We phenotyped 212 Italian families for PCOS following the Rotterdam diagnostic criteria (presence of at least two of the following: chronic anovulation or oligomenorrhea, clinical or biochemical hyperandrogenism, and/or polycystic ovaries) [36]. The families were originally recruited for T2D and descended from at least 3 generations of Italians. The Helsinki declarations guidelines were followed, and informed consent was obtained from each participant before enrollment in the study. The Bios Ethical Committee approved the study.

We genotyped via microarray 36 variants within the *CRHR1* gene and 18 variants within the *CRHR2* gene. After Mendelian and genotyping errors exclusion with PLINK [37], the variants were analyzed for parametric linkage to and/or linkage disequilibrium (LD, that is association) with PCOS using the models recessive with complete penetrance (R1) and dominant with complete penetrance (D1). In a secondary analysis, we tested the models dominant with incomplete penetrance (D2) and recessive with incomplete penetrance (R2). Variants with *p* of < 0.05 were considered statistically significant. We inferred the presence of LD blocks (correlation coefficient of ≥ 0.9) using the LD matrix of the Tuscany Italian population derived from the 1000 Genomes Project (https://www.internationalgenome.org/data-portal/population/TSI).

### In silico analysis

We analyzed the significant variants with various in silico tools that predict their role in transcription factor (TF) binding (SNP Function Prediction) [38], splicing (SpliceAI) [39], miRNA binding (mirSNP) [40] and regulatory potential (RegulomeDB) [28].

## Data Availability

The study data are available on reasonable request, and due to lacking specific patients’ consent and privacy restrictions, they are not publicly available.
